# Intravitreal aflibercept for diabetic macular edema in real-world clinical practice in Japan: 24-month outcomes

**DOI:** 10.1007/s00417-022-05703-9

**Published:** 2022-06-02

**Authors:** Masahiko Sugimoto, Chiharu Handa, Kazufumi Hirano, Toshiyuki Sunaya, Mineo Kondo

**Affiliations:** 1 Department of Ophthalmology, Mie University Graduate School of Medicine, 2-174 Edobashi, Tsu, Mie 514-8507 Japan; 2 Medical Affairs & Pharmacovigilance, Bayer Yakuhin, Ltd., Osaka, Japan; 3 Research & Development Japan, Bayer Yakuhin, Ltd., Osaka, Japan

**Keywords:** Diabetic macular edema, Intravitreal aflibercept, Anti-vascular endothelial growth factor treatment, Real-world clinical practice

## Abstract

**Purpose:**

To report the safety and effectiveness of intravitreal aflibercept (IVT-AFL) for diabetic macular edema (DME) in the real-world clinical practice setting in Japan.

**Methods:**

In this prospective, multicenter, observational, post-marketing surveillance, patients with DME newly receiving IVT-AFL were enrolled. During a 24-month follow-up, the primary outcome was the occurrence of safety events. Other pre-specified endpoints were effectiveness indicators, such as best-corrected visual acuity (BCVA), central retinal thickness, and injection frequency.

**Results:**

In total, 646 patients administered at least one IVT-AFL injection were included in the safety analysis. During the follow-up period, adverse events occurred in 42 patients (6.50%), whereas adverse drug reactions occurred in 12 (1.86%). In the 12 patients who had adverse drug reactions, seven events occurred in seven patients within the first month of the most recent injection. In addition, 622 patients were included in the effectiveness analysis set. The number of injections over 24 months was 3.6 ± 3.0 (mean ± standard deviation [*SD*]). BCVA (logarithm of the minimum angle of resolution) was 0.437 ± 0.362 (mean ± SD) (*n* = 622) at baseline and 0.321 ± 0.348 (*n* = 177) after 24 months of treatment with IVT-AFL. Central retinal thickness was 440.8 ± 134.2 μm (mean ± SD) (*n* = 444) at baseline and 355.5 ± 126.4 μm (*n* = 140) at 24 months.

**Conclusion:**

Routine administration of IVT-AFL for DME was not associated with new safety concerns, and BCVA outcomes were maintained over 24 months in the real-world setting. Nonetheless, patients in this real-world setting received fewer injections than those in clinical trials, suggesting that a margin for improvement exists in clinical practice.

**Trial registration:**

ClinicalTrials.gov: NCT02425501. 
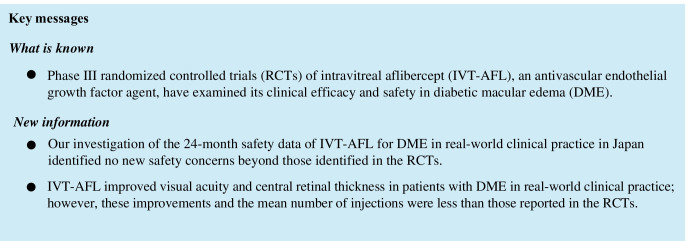

**Supplementary Information:**

The online version contains supplementary material available at 10.1007/s00417-022-05703-9.

## Introduction

Diabetic macular edema (DME) can occur at any stage of diabetic retinopathy and is the predominant cause of vision loss in patients with diabetes [1]. According to cross-sectional and population-based studies, the prevalence of DME ranges from 4.2 to 7.9% in patients with type 1 diabetes and from 1.4 to 12.8% in those with type 2 diabetes [2]. The main pathogenic elements in DME are the disruption of the blood–retinal barrier and vascular leakage, in which vascular endothelial growth factor (VEGF) plays a central role. Hypoxia, hyperglycemia, advanced glycation end products, inflammatory cytokines, and various growth factors increase VEGF expression, and excess VEGF leads to blood–retinal barrier disruption [3].

The treatment options for DME in Japan are intravitreal anti-VEGF therapy, laser photocoagulation, intravitreal steroid therapy, and vitrectomy [4]. Anti-VEGF therapy was associated with better visual improvement compared with photocoagulation in patients with DME in the RESTORE and VIVID/VISTA studies [5, 6], and 81.2% of ophthalmologists in Japan chose intravitreal anti-VEGF therapy as the first-line treatment for DME according to a survey conducted in 2016 and 2017 [7].

The anti-VEGF agent aflibercept has higher binding affinity for VEGF-A than that of its native receptors and also binds to VEGF-B and placental growth factor [8]. As evidence indicates that high intraocular concentrations of VEGF and placental growth factor are present in patients with DME [9–11], the efficacy of aflibercept has been hypothesized to be related to its binding properties. Additionally, the neutralizing efficacy of aflibercept against galectin-1, an angiogenic factor associated with diabetic retinopathy independent of VEGF-A, has been demonstrated [12]. The clinical efficacy of intravitreal aflibercept (IVT-AFL) in patients with DME was evaluated as the 12-month primary outcome in two randomized controlled trials (RCTs)—VIVID and VISTA—in which patients received either (i) IVT-AFL every 4 or 8 weeks after five initial monthly doses or (ii) laser photocoagulation. The results of these studies showed the superiority of IVT-AFL over laser photocoagulation in terms of the change from baseline in best-corrected visual acuity (BCVA) [5]. The safety profile of IVT-AFL in a Japanese population with DME was evaluated in VIVID-Japan, and the outcomes were similar to those observed in a non-Japanese patient population [13]. In Japan, IVT-AFL is currently approved for neovascular age-related macular degeneration, macular edema secondary to retinal vein occlusion, DME, choroidal neovascularization secondary to pathologic myopia, and neovascular glaucoma.

Due to the invasive nature of the intraocular injections, a safety protocol must be followed for the intravitreal injection of anti-VEGF agents. The main concern is that infectious organisms could potentially be introduced into the vitreous chamber, resulting in intraocular inflammatory conditions such as endophthalmitis. Mechanical issues associated with the injection procedure and pharmacodynamic-related local or systemic effects are also important [14]. The risk of sterile intraocular inflammation after anti-VEGF agent injection is a growing concern [15]. Major risks, which were selected with reference to phase III trials of IVT-AFL [5, 13, 16–20], include arterial thromboembolic events, traumatic cataract, retinal tear and detachment, increased intraocular pressure, and an intraocular inflammatory response. Information is lacking with regard to other events that could occur when IVT-AFL is combined with panretinal photocoagulation.

Accordingly, in this study, we assessed the safety data of IVT-AFL treatment in patients with DME in the real-world clinical practice setting in Japan and report our 24-month data on the effectiveness and safety of IVT-AFL. To our knowledge, this is the first report of a prospective observational study in a large number of patients with DME who received IVT-AFL in Japan.

## Methods

### Study design

This prospective, multicenter, observational, post-marketing surveillance was conducted in patients with DME in Japan from November 2014 to April 2019. A total of 134 physicians from 78 facilities participated in this surveillance. An electronic data capture system with a central registration method was used for the survey component, which had a maximum period of 24 months. Study physicians registered patients, confirmed the initial injections, and entered the results at the end of treatment in usual clinical practice. Patient observation was terminated if no further IVT-AFL treatment was planned after any of the following events occurred: (1) adverse event (AE)–related IVT-AFL discontinuation, insufficient therapeutic effect, or any other reason, or (2) loss to follow-up (loss of contact/no further visits).

A sample size of 300 patients was required to detect at least one AE occurring with an incidence of 1%, such as an arterial thromboembolic event, at a 95% probability. Furthermore, given that about 50% of patients with DME receive combination therapy with photocoagulation, steroids, or another treatment, the number of patients in the study was set to 600 to detect the aforementioned events in IVT-AFL monotherapy.

### Patients and treatment

Patients with a diagnosis of DME were enrolled after the investigator decided to initiate treatment with IVT-AFL. Patients who had previously been treated with IVT-AFL were excluded. The eye that received the first dose of IVT-AFL was included in the analysis. If both eyes were treated with IVT-AFL, the eye that started treatment earlier was selected for analysis; if treatment began in both eyes on the same day, the eye with the worse visual acuity at baseline was selected.

Prior treatments for DME were recorded as variables for patients who had received photocoagulation, surgery, another therapy, or an anti-VEGF agent other than IVT-AFL. After the first injection, the treating physician determined the need for repeat injections. The inter-injection interval was at least 4 weeks, in line with package insert guidelines.

### Outcome measures

Primary outcomes included the occurrence of all adverse drug reactions (ADRs; an AE deemed by the treating clinician to be definitely related to IVT-AFL), all infections, and ocular AEs and serious AEs (SAEs) for which a relationship to the intravitreal injection procedure could not be entirely excluded. These events were categorized according to the Medical Dictionary for Regulatory Activities version 22.0, using its terminology for each type of event. ADR occurrence based on patient characteristics was summarized as other pre-specified outcomes.

In line with the risk management plan mandated by the Japanese Pharmaceutical and Medical Devices Agency, safety specifications comprising major risks were also examined. These specifications are detailed in Supplementary Information 1.

Effectiveness variables included other pre-specified outcomes, including BCVA (logarithm of the minimum angle of resolution [logMAR]) and central retinal thickness (CRT) in the entire study population and in the subgroups (based on the presence or absence of previous treatment, baseline decimal BCVA [≤ 0.5, > 0.5]), as well as the proportion of patients with improved or maintained BCVA.

The treatment status data for patients were also summarized, including the number of injections, duration of the observation period, treatment continuation rate, and reasons for treatment discontinuation.

### Statistical analysis

The statistical analyses were exploratory and descriptive. Categorical variables are summarized as frequencies and proportions. Continuous variables are expressed as descriptive statistics. All statistical analyses were performed using SAS version 9.4 (SAS Institute Inc., Cary, NC).

Patients who received at least one IVT-AFL dose were included in the safety analysis set (SAS). The numbers and frequencies of patients who developed ADRs, serious ADRs, AEs, and SAEs for which a relationship to the intravitreal injection procedure could not be entirely excluded were summarized along with the safety specifications for the entire period, from the initial IVT-AFL to 24 months or every 30 days. Based on the classification of patient characteristics, the ADR incidence and 95% confidence interval (CI) were calculated. The time of onset of ADRs after the most recent IVT-AFL was summarized as a post hoc analysis.

The effectiveness analysis set (EAS) included patients who had at least one ophthalmologic evaluation before and after the first IVT-AFL injection. The analysis was performed using observed data, that is, the results were summarized only for patients for whom data at each evaluation point could be collected. BCVA and CRT were summarized over time. Changes in BCVA and CRT from baseline to 24 months were calculated. The numbers of injections in patients with both baseline and 24-month data were also calculated as a post hoc analysis. BCVA change from baseline was classified into the following three categories, with the proportions of patients in the categories calculated over time: “improved,” logMAR BCVA change ≤  − 0.3; “maintained,” logMAR BCVA change between − 0.3 and 0.3; or “worsened,” logMAR BCVA change ≥ 0.3.

Most facilities in Japan use decimal visual acuity for BCVA measurements. When summarizing BCVA in this analysis, decimal BCVA was converted to logMAR BCVA by the following formula: logMAR BCVA =  − log_10_(decimal BCVA). If the recorded decimal BCVA was 0.01 or less, it was converted to logMAR 2.00.

## Results

### Patient population

**Fig. 1:**
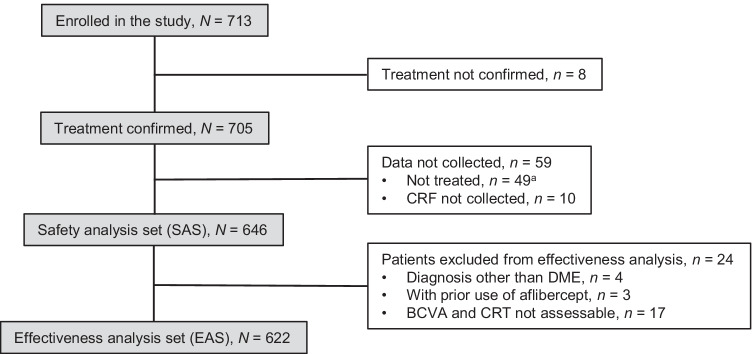
Patient flow diagram. *BCVA* best-corrected visual acuity, *CRF* case report form, *CRT* central retinal thickness, *DME* diabetic macular edema, *IVT-AFL* intravitreal aflibercept. ^a^Includes one patient with no recorded use of IVT-AFL on the CRF

In total, the present surveillance enrolled 713 patients; 646 received at least one IVT-AFL injection and were thus included in the SAS. After the exclusion of patients with diagnoses other than DME, patients with a history of IVT-AFL, and patients whose BCVA and CRT could not be assessed, data for 622 patients were included in the EAS (Fig. 1).

Table 1 presents the baseline patient characteristics in the SAS. The mean age was 64.9 years, and 62.7% were male. Decimal BCVAs at baseline were ≤ 0.5 in 410 patients (63.5%) and > 0.5 in 236 (36.5%), and the logMAR BCVA was 0.441 ± 0.364 (mean ± standard deviation, *n* = 646). CRT was 441.2 ± 134.6 μm (*n* = 453). In addition, 166 patients (25.7%) had no treatment history for DME, while 471 (72.9%) had received previous treatment. Prior treatment, medical history, and combination therapies are detailed in Supplementary Information 2–4, respectively.

**Table 1:** Patient characteristics at baseline

	Safety analysis set (*n* = 646)
Male, *n* (%)	405 (62.7)
Age, years	
Mean ± *SD*	64.9 ± 11.2
Median (range)	66.0 (26–89)
Stage of diabetic retinopathy, *n* (%)	
Simple diabetic retinopathy	156 (24.1)
Pre-proliferative diabetic retinopathy	275 (42.6)
Proliferative diabetic retinopathy	173 (26.8)
Unknown	42 (6.5)
Duration of diabetes mellitus, years, *n* (%)	
< 5	26 (4.0)
≥ 5, < 10	61 (9.4)
≥ 10	196 (30.3)
Unknown	363 (56.2)
HbA1c, *n* (%)	
≤ 7.0%	65 (10.1)
> 7.0%	68 (10.5)
Unknown	513 (79.4)
Extent of edema, *n* (%)	
Diffuse	417 (64.6)
Localized	194 (30.0)
Other	1 (0.2)
Unknown	34 (5.3)
Best-corrected visual acuity, logMAR	
Mean ± *SD*	0.441 ± 0.364
Median (range)	0.349 (− 0.08 to 2.00)
Best-corrected visual acuity, decimal, *n* (%)	
≤ 0.5 (logMAR ≤ 0.3)	410 (63.5)
> 0.5	236 (36.5)
Central retinal thickness, μm [*n* = 453]	
Mean ± *SD*	441.2 ± 134.6
Median (range)	432.0 (108–869)
Prior treatment, *n* (%)	
No	166 (25.7)
Yes	471 (72.9)
Unknown	9 (1.4)
Medical history	
Ocular	270 (41.8)
Non-ocular	135 (20.9)
Combination therapies, *n* (%)	
No	434 (67.2)
Yes	201 (31.1)
Unknown	11 (1.7)

*LogMAR* logarithm of the minimum angle of resolution, *SD* standard deviation

In the SAS, ocular comorbidities were reported in 209 patients (32.4%) at baseline, including cataract in 165 (25.5%), glaucoma in 38 (5.9%), and ocular hypertension and conjunctivitis in 9 (1.4%) each. Non-ocular comorbidities were reported in 222 patients (34.4%) and included hypertension in 159 (24.6%), renal impairment in 66 (10.2%), and hypercholesterolemia in 57 (8.8%) (Supplementary Information 5).

### Safety outcomes

In the SAS, the duration of observation was 565.8 ± 324.3 days (median, 700.5; range, 1–1,437) and the numbers of injections were 2.3 ± 1.3 up to 6 months, 2.9 ± 2.1 up to 12 months, and 3.5 ± 3.0 (median, 3.0; range, 1–18) up to 24 months. In addition, 202 patients (31.3%) received just one dose of IVT-AFL.

In the SAS, 42 patients (6.50%) had AEs and 24 (3.72%) had SAEs (Table 2). AEs observed in three or more patients included vitreous hemorrhage in five patients (0.77%), cataract, increased intraocular pressure and cerebral infarction in four patients (0.62%), and allergic conjunctivitis and chronic kidney disease in three patients (0.46%). SAEs observed in three or more patients included cerebral infarction in four patients (0.62%) and chronic kidney disease in three (0.46%) (Supplementary Information 6).

**Table 2:** Incidence of ocular and non-ocular adverse events and adverse drug reactions

Safety analysis set (*n* = 646)	Patients, *n* (%)			
	AE	SAE	ADR	SADR
Total events	42 (6.50)^a^	24 (3.72)^a^	12 (1.86)	7 (1.08)
Ocular events	27 (4.18)^a^	9 (1.39)^a^	8 (1.24)	4 (0.62)
Non-ocular events	18 (2.79)^a^	15 (2.32)^a^	4 (0.62)	3 (0.46)

*AE* adverse event, *SAE* serious adverse event, *ADR* adverse drug reaction, *SADR* serious adverse drug reaction

^a^Including patients with multiple events

Adverse drug reactions occurred in 12 patients (1.86%) in the SAS: three (0.46%) with cataract, two (0.31%) with cerebral infarction, and one (0.15%) each with retinal artery occlusion, tractional retinal detachment, eye pain, increased intraocular pressure, lenticular injury, facial paralysis, and myocardial infarction. There were no reports of infection. Serious ADRs occurred in seven patients (1.08%): two (0.31%) with cerebral infarction and one (0.15%) each with cataract, retinal artery occlusion, tractional retinal detachment, lenticular injury, and myocardial infarction (Table 2; Supplementary Information 6). The cases of cerebral infarction, retinal artery occlusion, and tractional retinal detachment all recovered, while the cases of cataract, myocardial infarction, and lenticular injury were recovering as of the last follow-up visit.

As AEs for which a relationship to the intravitreal injection procedure could not be entirely excluded, retinal artery occlusion and lenticular injury were observed in one patient each, and both were serious. The retinal artery occlusion recovered with anterior chamber paracentesis and eye massage. The lenticular injury was suspected to be related to concomitant drug treatment (cefcapene pivoxil, fluorometholone, bromfenac sodium, and betamethasone) rather than IVT-AFL, and the patient was recovering following cataract surgery.

Of the 12 patients with ADRs, seven had the events within 1 month of the most recent IVT-AFL injection and 11 had the events within 2 months. Six patients with eye disorders experienced the events within 2 months and four experienced the events within 1 month. The ADR with the longest time to onset was myocardial infarction, which occurred 5 months after the last IVT-AFL injection. Of the two cerebral infarctions, one occurred within 1 month of the most recent IVT-AFL injection and the other occurred within 2 months.

Based on each patient characteristic, overlaps of the 95% CIs of the incidence proportion were found for all stratification factors, suggesting that no patient factors affected the occurrence of ADRs.

Safety specifications defined by the risk management plan and observed as ADRs included increased intraocular pressure (*n* = 1), retinal tear and retinal detachment (*n* = 1), traumatic cataract (*n* = 1), arterial thromboembolic events (*n* = 3), and ADRs that occurred when IVT-AFL was used in combination with panretinal photocoagulation (*n* = 1). No events corresponding to an intraocular inflammatory response were reported. All arterial thromboembolic ADRs (cerebral infarction in two patients and myocardial infarction in one) were serious and led to discontinuation of treatment with IVT-AFL (Table 3). The two patients with cerebral infarction were ≥ 70 years of age, and they both recovered; one had type 2 diabetes mellitus and hypertension as risk factors other than IVT-AFL. The patient with myocardial infarction was ≥ 60 years of age; the patient was recovering and had comorbidities suspected to be risk factors other than IVT-AFL.

**Table 3:** Safety specifications: incidence of adverse events and adverse drug reactions

Safety analysis set (*n* = 646)	Patients, *n* (%)			
	Adverse event		Adverse drug reaction	
	Serious	Non-serious	Serious	Non-serious
Important risks identified				
Inflammatory intraocular response	0	0	0	0
Increased intraocular pressure	1 (0.15)	4 (0.62)	0	1 (0.15)
Retinal tear and retinal detachment	2 (0.31)	0	1 (0.15)	0
Traumatic cataract	1 (0.15)	0	1 (0.15)	0
Important potential risk				
Arterial thromboembolic events	5 (0.77)	0	3 (0.46)	0
Important information deficiency				
Events that occurred when used in combination with PRP	10 (1.55)		1 (0.15)	0

Medical Dictionary for Regulatory Activities terms for each type of event are listed in Supplementary Information 1

*PRP* panretinal photocoagulation

### Effectiveness outcomes

#### Treatment status

In the complete EAS (*n* = 622), the duration of observation was 566.6 ± 320.8 days (median, 701.5; range, 10–1437), and the numbers of injections were 2.3 ± 1.4 up to 6 months, 2.9 ± 2.1 up to 12 months, and 3.6 ± 3.0 (median, 3.0; range, 1–18) up to 24 months. In addition, 186 patients (29.9%) received just one dose of IVT-AFL.

Treatment continuation rates at 6, 12, and 24 months were 80.7%, 66.6%, and 53.7%, respectively. The principal reasons given for discontinuation (including multiple answers) were achievement of the treatment goal (*n* = 73; 25.3%), visit cessation (*n* = 62; 21.5%), and referral to another hospital (*n* = 60; 20.8%). Nine patients (3.1%) discontinued treatment due to AEs.

#### Visual acuity

**Fig. 2:**
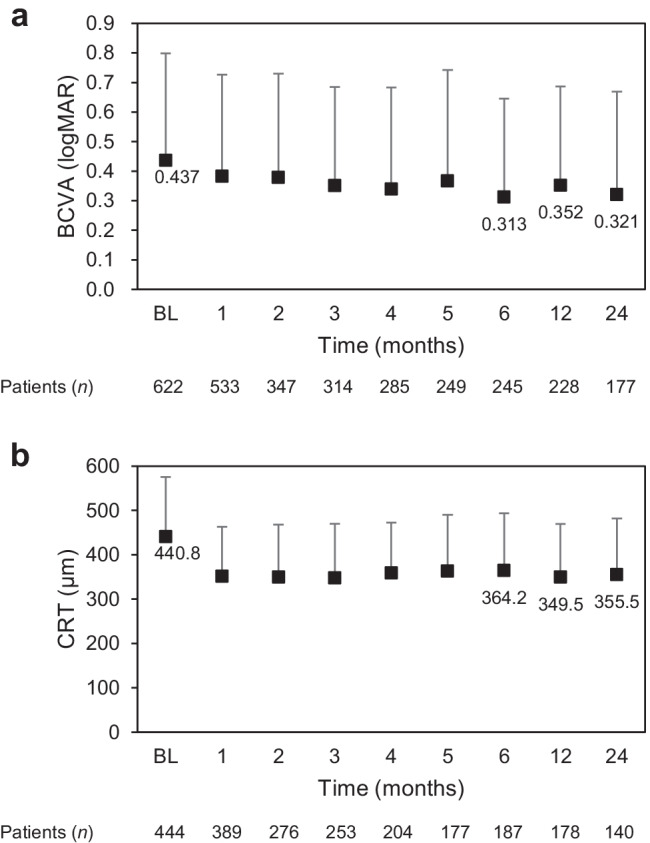
**a** LogMAR BCVAs and numbers of patients during the 24-month study period. Data are not shown for eight patients whose previous treatment status was unknown. **b** CRTs (μm) and numbers of patients during the 24-month study period. Data are not shown for three patients whose previous treatment status was unknown. The mean and standard deviation are indicated with markers and whiskers, respectively. *BCVA* best-corrected visual acuit, *BL* baseline, *CRT* central retinal thickness, *logMAR* logarithm of the minimum angle of resolution

LogMAR BCVAs were 0.437 ± 0.362 (median, 0.301; range, − 0.08 to 2.00) at baseline and 0.321 ± 0.348 (median, 0.222; range, − 0.18 to 2.00) after 24 months of IVT-AFL treatment (Fig. 2a). The logMAR BCVA change from baseline to 24 months was − 0.071 ± 0.323 (median, − 0.079; range, − 1.48 to 1.12) with 5.4 ± 3.7 injections (median, 5.0; range, 1–18) (*n* = 177).

In the EAS, the proportion of patients with improved BCVA was approximately 15%, and the proportion of patients with improved or maintained BCVA was approximately 90% throughout the 24 months of IVT-AFL treatment. At 24 months, the statuses of 156 patients (88.1%) were considered to be improved or maintained (Supplementary Information 7).

#### Central retinal thickness

Central retinal thickness was 440.8 ± 134.2 μm (median, 432.0; range, 108–869) at baseline and 355.5 ± 126.4 μm (median, 326.0; range, 126–954) after 24 months of IVT-AFL treatment (Fig. 2b). The CRT change from baseline to 24 months was − 102.8 ± 146.4 μm (median, − 68.5; range, − 427 to 298) (*n* = 132).

#### Subgroup analysis based on previous treatment status

LogMAR BCVAs in the subgroups of patients with and without previous treatment were 0.454 ± 0.359 and 0.393 ± 0.372 at baseline and 0.360 ± 0.363 and 0.207 ± 0.266 at 24 months, respectively (Supplementary Information 8a). The logMAR BCVA changes from baseline to 24 months in the patients with and without previous treatment were − 0.035 ± 0.321 (median, − 0.046; range, − 1.48 to 1.12) with 5.6 ± 3.7 injections (median, 5.0; range, 1–18) (*n* = 132) and − 0.179 ± 0.318 (median, − 0.117; range, − 1.26 to 0.40) with 5.0 ± 3.8 injections (median, 4.5; range, 1–18) (*n* = 42), respectively.

In patients with and without previous treatment, CRTs were 446.2 ± 136.8 μm and 421.7 ± 122.8 μm at baseline and 357.8 ± 131.8 μm and 351.7 ± 106.0 μm at 24 months, respectively (Supplementary Information 8b). The CRT changes from baseline to 24 months in the patients with and without previous treatment were − 101.4 ± 149.5 μm (median, − 68.0; range, − 427 to 298; *n* = 101) and − 105.7 ± 139.9 μm (median, − 66.5; range, − 395 to 144; *n* = 30), respectively.

#### Subgroup analysis based on the decimal BCVA at baseline

In the subgroups of patients with baseline decimal BCVAs ≤ 0.5 and > 0.5, the logMAR BCVAs were 0.620 ± 0.333 and 0.123 ± 0.092 at baseline and 0.463 ± 0.364 and 0.119 ± 0.190 at 24 months, respectively (Supplementary Information 9a). The logMAR BCVA changes from baseline to 24 months in the patients with baseline decimal BCVAs ≤ 0.5 and > 0.5 were − 0.127 ± 0.385 (median, − 0.161; range, − 1.48 to 1.12) with 5.6 ± 3.9 injections (median, 5.0; range, 1–18) (*n* = 104) and 0.008 ± 0.179 (median, 0.000; range, − 0.40 to 0.78) with 5.1 ± 3.4 injections (median, 5.0; range, 1–18) (*n* = 73), respectively.

In the subgroups of patients with baseline decimal BCVAs ≤ 0.5 or > 0.5, CRTs were 464.0 ± 140.6 μm and 405.4 ± 115.6 μm at baseline and 349.8 ± 123.8 μm and 363.4 ± 130.5 μm at 24 months, respectively (Supplementary Information 9b). The CRT changes from baseline to 24 months in the patients with baseline decimal BCVAs ≤ 0.5 and > 0.5 were − 133.5 ± 147.8 μm (median, − 84.0; range, − 427 to 211; *n* = 75) and − 62.3 ± 135.3 μm (median, − 57.0; range, − 378 to 298; *n* = 57), respectively.

## Discussion

In the present analysis of the real-world safety profile of IVT-AFL in patients with DME, we found no new safety concerns beyond those identified in the VIVID/VISTA studies [5]. The incidence of ADRs in this 24-month study was 1.86% (12 of 646 patients), which was numerically lower than the 37.81% of patients (276 of 730, including non-Japanese individuals) reported in RCTs up to 12 months [21]. The incidence of intraocular inflammation based on the total number of IVT-AFL injections was 0.2 to 0.3% in the RCTs [22]. However, no intraocular inflammation was observed in this study. No patient factors affecting the occurrence of ADRs were observed.

Doses of anti-VEGF agents are higher when administered systemically than when delivered by intravitreal injection, and anti-VEGF therapy for the treatment of cancer is thought to be linked to a reduced production of nitric oxide and prostacyclin and increased production of erythropoietin, both of which can increase the risk of arterial thromboembolic events [23]. In this study, the information on arterial thromboembolic events was collected as one of the safety specifications, and thrombotic SAEs occurred in five patients. However, in the post hoc analysis in DRCR.net Protocol T, which compared the intravitreal injections of bevacizumab, ranibizumab, and aflibercept in patients with DME, there were no significant associations between the plasma levels of free VEGF and the incidence of systemic AEs, although it is possible that intravitreal injection of anti-VEGF agents may reduce the plasma level of free VEGF [24]. Additionally, in patients with retinal diseases, systemic pharmacokinetic/pharmacodynamic analysis of IVT-AFL found that it had no effects on blood pressure, a sensitive indicator of systemic VEGF inhibition [25].

Based on these safety results with IVT-AFL, there are no new concerns requiring attention, suggesting that no specific measures need to be taken at this time. However, attention should be paid to ADRs, especially in the first few months following initiation of IVT-AFL treatment, regardless of the injection regimen and visit schedule, as 11 of the 12 patients with systemic or ocular local ADRs had events within 2 months of the most recent IVT-AFL injection.

In this 24-month study, IVT-AFL numerically improved the mean logMAR BCVA and CRT of patients with DME, and these values were lower throughout the study period than at baseline. Given that approximately 15% of patients had improved BCVA and approximately 90% had improved or maintained BCVA, a certain degree of effectiveness of IVT-AFL was observed even in this clinical practice setting. However, the treatment continuation proportion of the EAS population at 24 months was around 44%, suggesting that the long-term continuation of treatment in the clinical practice setting is difficult. In addition, because the mean value of the final decimal BCVA was less than 20/40 (= useful visual acuity [26]), it is possible that a satisfactory therapeutic effect might not have been obtained in clinical practice.

In the STREAT-DME study, which retrospectively investigated real-world therapeutic outcomes after a 24-month clinical intervention for DME in Japan, the mean 24-month changes in BCVA (logMAR) were − 0.09 ± 0.39 (4.3 ± 3.6 injections) in the anti-VEGF monotherapy group and − 0.02 ± 0.40 (3.6 ± 3.1 injections) in the anti-VEGF combination therapy group. Furthermore, the percentages of eyes with a final BCVA > 20/40 were 49.4% and 38.9% in the monotherapy and combination therapy groups, respectively [26]. Although the STREAT-DME study had different conditions from our study, such as targeting only treatment-naïve populations and including anti-VEGF agents other than aflibercept, there were no marked differences in the functional outcome and number of injections between the two studies. Accordingly, although the proportion of patients with improved or maintained BCVA was 90% or more throughout the 24 months of this study, nearly half of the patients did not achieve BCVA > 20/40 and a satisfactory visual acuity prognosis in clinical practice.

In the VIVID/VISTA studies, the BCVA changes over 24 months were + 9.4 to + 11.1 letters, and the CRT changes were − 195.8 to − 191.1 μm [22]. Although these values are higher than those in our study, there were differences in patient background and study design, making direct comparison of the results difficult. In the RCTs, the mean numbers of injections up to 100 weeks were 13.5 to 13.6 [22]; the difference in the injection number might have contributed to the difference in the therapeutic effect between the RCTs and this study, in which the mean number of injections up to 24 months was 3.6 ± 3.0 in the EAS.

In this study, the selection of treatment regimen was left to the physician’s discretion. In the questionnaire survey on anti-VEGF therapy for DME in clinical practice in Japan conducted in 2016 and 2017, 53.4% of physicians selected a single injection as the initial dose and 75.0% selected the *pro re nata* (PRN) regimen in the maintenance phase [7]. In another survey conducted in 2015, 51.4% of physicians continued the initial monthly doses until the CRT stabilized and 76.3% provided maintenance injections using a PRN regimen [27]. Therefore, it is highly possible that the 1 + PRN regimen was also predominant in our study. Thus, the injection numbers in this study may have been lower than in the RCTs because the criteria for repeat injection were not strictly defined and a reactive regimen was mainly adopted. It may be possible to achieve better visual improvement by adopting a proactive regimen such as treat and extend, which is supported by a retrospective study of treatment-naïve patients with DME. In that study, a 24-month treat and extend regimen of anti-VEGF treatment led to significant improvement in BCVA in 26 eyes [28].

In the subgroup analysis based on previous treatment status, there was no marked difference in the mean number of injections between subgroups; however, the subgroup without previous treatment tended to have a numerically better final BCVA and a higher degree of change in BCVA. These results suggest that better visual prognosis could perhaps be obtained by starting anti-VEGF therapy as the first-line treatment at an earlier disease stage in patients who are expected to respond to treatment.

The subgroup analysis based on the decimal BCVA at baseline also showed no marked difference in the mean number of injections between subgroups. The degree of change in BCVA tended to be numerically better in patients with a baseline decimal BCVA ≤ 0.5. However, the final logMAR BCVA was numerically lower in the patients with a baseline decimal BCVA > 0.5, and the mean BCVA at 24 months in this group was above the lower limit of driving vision (0.7 in the decimal scale). In the VIVID/VISTA studies, the baseline BCVA of the patients ranged from 20/40 to 20/320 [5], which indicated a similar population to the subgroup with a baseline decimal BCVA ≤ 0.5 in this study. However, better BCVA improvement was obtained in the RCTs. In addition, in the DRCR.net Protocol T, the aflibercept subgroup with at least moderate vision loss at baseline (BCVA < 20/50) obtained a mean BCVA improvement of 18.1 ± 13.8 letters over 24 months (median number of injections, 15) [29]. Therefore, in patients with poor visual acuity, a treatment with more frequent injections, similar to the clinical trials, might improve the therapeutic results in clinical practice.

The strengths of our study are its the prospective study design and the fact that it is the first observational study in Japan of more than 600 patients with DME who received IVT-AFL treatment. However, several limitations of our study also need to be considered. Diagnostic and examination decisions were left to the physicians’ discretion. The eligibility criteria did not consider the risk of AEs or ADRs; however, physicians may have done so and, thus, may have introduced a selection bias. In contrast to the prior RCTs, the length between follow-up appointments was not strictly controlled; therefore, some outcome data were missing at each evaluation point, necessitating careful interpretation of the cohort data. Additionally, statistical analysis of the effectiveness data was not performed.

This study was a 24-month prospective observational study investigating safety and effectiveness in patients with DME who started IVT-AFL treatment in clinical practice in Japan. The study period was from November 2014 to April 2019, and the sizes of the SAS and EAS populations were 646 and 622 patients, respectively. The present work identified no new issues regarding the safety of IVT-AFL treatment and the results indicate a certain degree of effectiveness, suggesting that a favorable risk–benefit balance of IVT-AFL treatment on DME is maintained under real-world clinical conditions. Given that events occurred within 2 months of the most recent IVT-AFL injection in 11 of the 12 patients with systemic or ocular local ADRs, clinicians should pay particular attention to ADRs in the first few months of IVT-AFL treatment, regardless of the injection regimen and visit schedule. From the gap in the improvement in BCVA and number of injections between RCTs and this study, there seems to be margin for improvement in the clinical treatment results, and subgroup analysis suggested that more frequent or proactive interventions from an earlier disease stage may provide better therapeutic effects.

## Supplementary Information

Below is the link to the electronic supplementary material.

Supplementary file1 (PDF 110 KB)

## Data Availability

Availability of the data underlying this publication will be determined in accordance with Bayer’s commitment to the EFPIA/PhRMA “Principles for responsible clinical trial data sharing.” This pertains to the scope, time point, and process of data access. As such, Bayer commits to sharing upon reasonable request from qualified scientific and medical researchers those patient-level clinical trial data, study-level clinical trial data, and protocols from clinical trials in patients for medicines and indications approved in the United States (US) and European Union (EU) as necessary for conducting legitimate research. This applies to data on new medicines and indications that have been approved by the EU and US regulatory agencies on or after January 01, 2014. Interested researchers can use www.clinicalstudydatarequest.com to request access to anonymized patient-level data and supporting documents from clinical studies to conduct further research that can help advance medical science or improve patient care. Information on the Bayer criteria for listing studies and other relevant information is provided in the “Study sponsors” section of the portal. Data access will be granted to anonymized patient-level data, protocols, and clinical study reports after approval by an independent scientific review panel. Bayer is not involved in the decisions made by the independent review panel. Bayer will take all necessary measures to ensure that patient privacy is safeguarded.
