# Correction to: Intravitreal aflibercept for diabetic macular edema in real‑world clinical practice in Japan: 24‑month outcomes

**DOI:** 10.1007/s00417-022-05917-x

**Published:** 2022-12-06

**Authors:** Masahiko Sugimoto, Chiharu Handa, Kazufumi Hirano, Toshiyuki Sunaya, Mineo Kondo

**Affiliations:** 1 Department of Ophthalmology, Mie University Graduate School of Medicine, 2‑174 Edobashi, Tsu, Mie 514‑8507 Japan; 2 Medical Affairs & Pharmacovigilance, Bayer Yakuhin, Ltd., Osaka, Japan; 3 Research & Development Japan, Bayer Yakuhin, Ltd., Osaka, Japan

**Correction to** **:** **Graefe’s Archive for Clinical and Experimental Ophthalmology (2022) 260:3489–3498**

10.1007/s00417-022-05703-9

This article contained some errors:

#1. In Table 1, the direction of the inequality sign in the logMAR Best-corrected visual acuity was reversed.

#2. Regarding Supplementary Information 4, the patients who received combination therapies were corrected in accordance with the communication with the Pharmaceuticals and Medical Devices Agency. Although there were additions in some categories, these additional patients also had received other combination therapies, thus there was no change in the total number of patients receiving the combination therapies.

#3. Regarding “Events that occurred when used in combination with PRP” in Table 3, the proportion of the number of patients who developed these events was originally calculated using the safety analysis set (n = 646) as a denominator, but this time, the number of the patients who received PRP (n = 81) as combination therapy was used as a denominator. In addition, we recounted the number of patients who developed these adverse events by distinguishing between serious and non-serious.

#4. In the footnote of Fig. 2, we had mistakenly put the text that should have been included in the footnote of Supplementary information 8.

The correct tables and legends are shown below. Revisions are shown in green.

**Table 1:** Patient characteristics at baseline

	Safety analysis set (*n* = 646)
Male, *n* (%)	405 (62.7)
Age, years	
Mean ± *SD*	64.9 ± 11.2
Median (range)	66.0 (26–89)
Stage of diabetic retinopathy, *n* (%)	
Simple diabetic retinopathy	156 (24.1)
Pre-proliferative diabetic retinopathy	275 (42.6)
Proliferative diabetic retinopathy	173 (26.8)
Unknown	42 (6.5)
Duration of diabetes mellitus, years, *n* (%)	
< 5	26 (4.0)
≥ 5, < 10	61 (9.4)
≥ 10	196 (30.3)
Unknown	363 (56.2)
HbA1c, *n* (%)	
≤ 7.0%	65 (10.1)
> 7.0%	68 (10.5)
Unknown	513 (79.4)
Extent of edema, *n* (%)	
Diffuse	417 (64.6)
Localized	194 (30.0)
Other	1 (0.2)
Unknown	34 (5.3)
Best-corrected visual acuity, logMAR	
Mean ± *SD*	0.441 ± 0.364
Median (range)	0.349 (− 0.08 to 2.00)
Best-corrected visual acuity, decimal, *n* (%)	
≤ 0.5 (logMAR 0.3)	410 (63.5)
> 0.5	236 (36.5)
Central retinal thickness, μm (*n* = 453)	
Mean ± SD	441.2 ± 134.6
Median (range)	432.0 (108–869)
Prior treatment, *n* (%)	
No	166 (25.7)
Yes	471 (72.9)
Unknown	9 (1.4)
Medical history	
Ocular	270 (41.8)
Non-ocular	135 (20.9)
Combination therapies, *n* (%)	
No	434 (67.2)
Yes	201 (31.1)
Unknown	11 (1.7)

*LogMAR* logarithm of the minimum angle of resolution, *SD* standard deviation

**Supplementary Information 4** Combination therapies^a^

**Table:** 

	Patients, n (%)^b^
Safety analysis set	646 (100)
Absence of combination therapies	434 (67.2)
Presence of combination therapies^a^	201 (31.1)
Panretinal photocoagulation	
Corticosteroids	
Surgery	52 (8.0)
Direct coagulation	45 (7.0)
Grid coagulation	3 (0.5)
Other	10 (1.5)

^a^Drug treatment other than IVT-AFL, photocoagulation or surgery, performed for DME after the first dose of IVT-AFL

^b^Counted under all applicable categories

Table 3

**Table 3:**
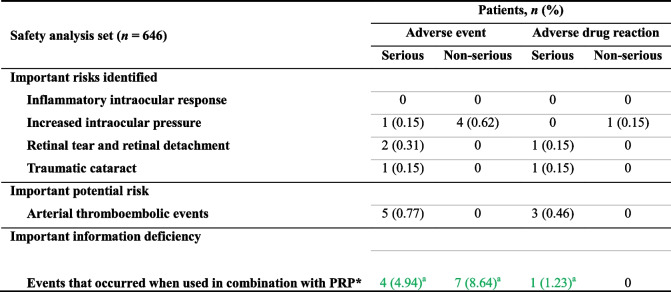
Safety specifications: incidence of adverse events and adverse drug reactions

Medical Dictionary for Regulatory Activities terms for each type of event are listed in Supplementary Information 1

*PRP* panretinal photocoagulation

*Including patients with multiple events

Figure 2

**Fig. 2:**
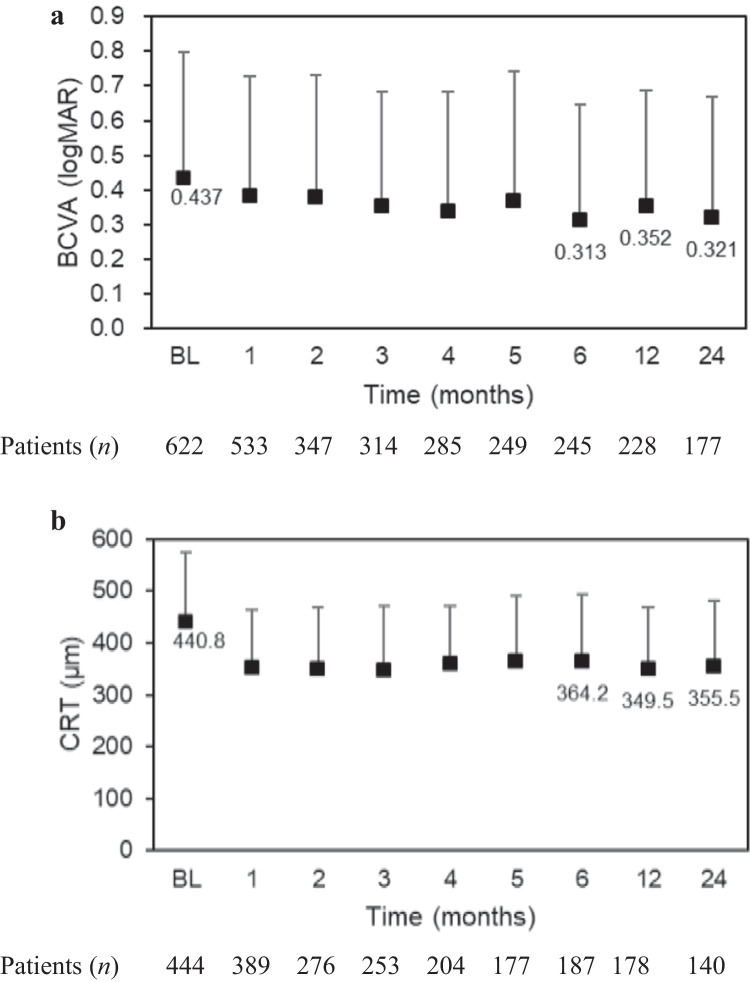
**a** LogMAR BCVAs and numbers of patients during the 24-month study period. **b** CRTs (μm) and numbers of patients during the 24-month study period. The mean and standard deviation are indicated with markers and whiskers, respectively. *BCVA* best-corrected visual acuity, *BL* baseline, *CRT* central retinal thickness, *logMAR* logarithm of the minimum angle of resolution


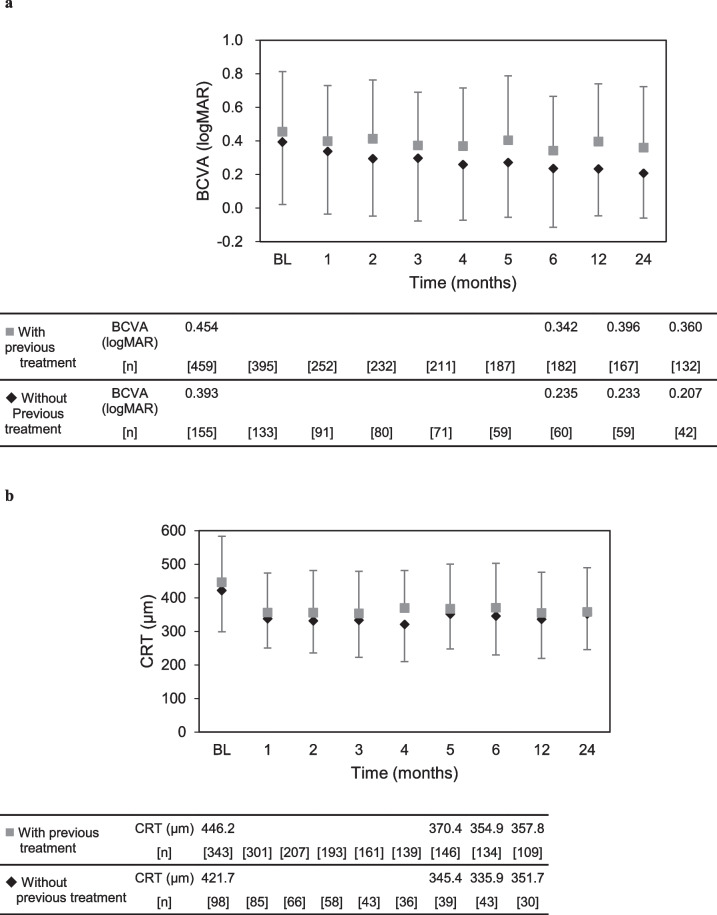
**Supplementary Information 8** Subgroup analysis based on the presence/absence of previous treatment. (a) LogMAR BCVAs and numbers of patients during the 24-month study period.  (b) CRTs (μm) and numbers of patients during the 24-month study period.  The mean and standard deviation are indicated with markers and whiskers, respectively.

*BCVA* best-corrected visual acuity; *BL* baseline; *CRT* central retinal thickness; *logMAR* logarithm of the minimum angle of resolution

