# Supplementary material for: Intravitreal aflibercept for diabetic macular edema in real-world clinical practice in Japan: 24-month outcomes
Source: Graefes Arch Clin Exp Ophthalmol. 2022 Jun 2;260(11):3489–98. doi: 10.1007/s00417-022-05703-9 (PMC9581854; doi:10.1007/s00417-022-05703-9)
Supplement: Supplementary file 1 — Supplementary file1 (PDF 110 KB) [file 417_2022_5703_MOESM1_ESM.pdf]

**Title**

# Intravitreal aflibercept for diabetic macular edema in real-world clinical practice in Japan: 24-month outcomes

**Journal**

Graefe's Archive for Clinical and Experimental Ophthalmology

**Authors**

Masahiko Sugimoto,[1*] Chiharu Handa,[2] Kazufumi Hirano,[2] Toshiyuki Sunaya,[3] and Mineo Kondo[1]

[1]Department of Ophthalmology, Mie University Graduate School of Medicine, Mie, Japan

[2]Medical Affairs & Pharmacovigilance, Bayer Yakuhin, Ltd., Osaka, Japan

[3]Research & Development Japan, Bayer Yakuhin, Ltd., Osaka, Japan

*Corresponding author: Masahiko Sugimoto, MD, PhD

Department of Ophthalmology

Mie University Graduate School of Medicine

2-174 Edobashi, Tsu, Mie 514-8507, Japan

Phone: +81-59-231-5027, Fax: +81-59-231-3036

E-Mail: sugmochi@clin.medic.mie-u.ac.jp

**Supplementary Information 1** Safety specification definitions

| | |
|---|---|
| **Important risks identified** | |
| **Intraocular inflammatory response** | Anterior chamber cell, anterior chamber fibrin, anterior chamber flare, anterior chamber inflammation, aqueous fibrin, autoimmune uveitis, candida endophthalmitis, choroiditis, chorioretinitis, cyclitis, endophthalmitis, eye infection, eye infection bacterial, eye infection chlamydial, eye infection fungal, eye infection intraocular, eye infection staphylococcal, eye inflammation, hypopyon, infectious iridocyclitis, infective iritis, infective uveitis, iridocyclitis, iritis, mycotic endophthalmitis, non-infectious endophthalmitis, noninfective chorioretinitis, pseudoendophthalmitis, uveitis, vitreal cells, vitreous fibrin, vitritis, necrotizing retinitis |
| **Increased intraocular pressure** | Intraocular pressure increased, ocular hypertension |
| **Retinal tear and retinal detachment** | Macular detachment, retinal tear, retinal detachment, rhegmatogenous retinal detachment, serous retinal detachment, tractional retinal detachment |
| **Traumatic cataract** | Atopic cataract, cataract, cataract cortical, cataract diabetic, cataract nuclear, cataract operation, cataract subcapsular, cataract traumatic, intraocular lens implant, lens capsulotomy, lens discoloration, lens extraction, lenticular injury, lenticular opacities, lenticular operation, posterior lens capsulotomy, radiation cataract, toxic cataract |
| **Important potential risk** | |
| **Arterial thromboembolic events** | [SMQ] Ischemic central nervous system vascular conditions (SMQ) [Narrow]<br>[SMQ] Ischemic heart disease (SMQ) [Broad] |
| **Important information deficiency** | |
| **Events that occurred when used in combination with PRP** | – |

*MedDRA* Medical Dictionary for Regulatory Activities; *PRP* panretinal photocoagulation; *SMQ* standardized MedDRA queries

**Supplementary Information 2** Prior treatments

| | Patients, n (%)[a] |
|---|---|
| **Presence of prior treatment** | 471 (100) |
| **Panretinal photocoagulation** | 357 (75.8) |
| **Corticosteroids** | 184 (39.1) |
| **Other anti-VEGF agents** | 116 (24.6) |
| **Direct coagulation** | 75 (15.9) |
| **Surgery** | 58 (12.3) |
| **Grid coagulation** | 9 (1.9) |
| **Other** | 5 (1.1) |

*VEGF* vascular endothelial growth factor

[a]Counted under all applicable categories

**Supplementary Information 3** Medical history

| | Patients, *n* (%)[a] |
|---|---|
| **Safety analysis set** | 646 (100) |
| **Ocular** | 270 (41.8) |
| **Cataract** | 254 (39.3) |
| **Ocular hypertension** | 8 (1.2) |
| **Conjunctivitis** | 8 (1.2) |
| **Glaucoma** | 6 (0.9) |
| **Uveitis** | 2 (0.3) |
| **Other** | 17 (2.6) |
| **Non-ocular** | 135 (20.9) |
| **Hypertension** | 51 (7.9) |
| **Renal impairment** | 26 (4.0) |
| **Hypercholesterolemia** | 16 (2.5) |
| **Old cerebral infarction** | 16 (2.5) |
| **Angina pectoris** | 8 (1.2) |
| **Old myocardial infarction** | 6 (0.9) |
| **Hypertriglyceridemia** | 5 (0.8) |
| **Acute myocardial infarction** | 3 (0.5) |
| **Impaired liver function** | 2 (0.3) |
| **Cerebral hemorrhage** | 2 (0.3) |
| **Subarachnoid hemorrhage** | 1 (0.2) |
| **Other** | 49 (7.6) |

[a]Counted under all applicable categories

**Supplementary Information 4** Combination therapies[a]

| | Patients, n (%)[b] |
|---|---|
| **Safety analysis set** | 646 (100) |
| **Absence of combination therapies** | 434 (67.2) |
| **Presence of combination therapies**[a] | 201 (31.1) |
| **Panretinal photocoagulation** | 80 (12.4) |
| **Corticosteroids** | 55 (8.5) |
| **Surgery** | 52 (8.0) |
| **Direct coagulation** | 45 (7.0) |
| **Grid coagulation** | 3 (0.5) |
| **Other** | 10 (1.5) |

[a]Drug treatment other than IVT-AFL, photocoagulation or surgery, performed for DME after the first dose of IVT-AFL

[b]Counted under all applicable categories

**Supplementary Information 5** Comorbidities at baseline

| | Patients, *n* (%)[a] |
|---|---|
| **Safety analysis set** | 646 (100) |
| **Ocular** | 209 (32.4) |
| **Cataract** | 165 (25.5) |
| **Glaucoma** | 38 (5.9) |
| **Ocular hypertension** | 9 (1.4) |
| **Conjunctivitis** | 9 (1.4) |
| **Retinal pigment epitheliopathy** | 2 (0.3) |
| **Other** | 23 (3.6) |
| **Non-ocular** | 222 (34.4) |
| **Hypertension** | 159 (24.6) |
| **Renal impairment** | 66 (10.2) |
| **Hypercholesterolemia** | 57 (8.8) |
| **Hypertriglyceridemia** | 21 (3.3) |
| **Angina pectoris** | 12 (1.9) |
| **Impaired liver function** | 7 (1.1) |
| **Old cerebral infarction** | 3 (0.5) |
| **Old myocardial infarction** | 1 (0.2) |
| **Other** | 47 (7.3) |

[a]Counted under all applicable categories

**Supplementary Information 6** Incidence of adverse events and adverse drug reactions

| Safety analysis set (n =646) | Patients, *n* (%) | | | |
|---|---|---|---|---|
| | AE | SAE | ADR | SADR |
| **Total events** | 42 (6.50)[a] | 24 (3.72)[a] | 12 (1.86) | 7 (1.08) |
| **Ocular events, total** | 27 (4.18)[a] | 9 (1.39)[a] | 8 (1.24) | 4 (0.62) |
| **Eye disorders** | 22 (3.41)[a] | 7 (1.08) | 6 (0.93) | 3 (0.46) |
| Vitreous hemorrhage | 5 (0.77) | 0 | 0 | 0 |
| Cataract | 4 (0.62) | 2 (0.31) | 3 (0.46) | 1 (0.15) |
| Conjunctivitis allergic | 3 (0.46) | 0 | 0 | 0 |
| Glaucoma | 2 (0.31) | 1 (0.15) | 0 | 0 |
| Posterior capsule opacification | 2 (0.31) | 0 | 0 | 0 |
| Retinal artery occlusion | 1 (0.15) | 1 (0.15) | 1 (0.15) | 1 (0.15) |
| Tractional retinal detachment | 1 (0.15) | 1 (0.15) | 1 (0.15) | 1 (0.15) |
| Eye pain | 1 (0.15) | 0 | 1 (0.15) | 0 |
| Iris adhesions | 1 (0.15) | 1 (0.15) | 0 | 0 |
| Retinal detachment | 1 (0.15) | 1 (0.15) | 0 | 0 |
| Asthenopia | 1 (0.15) | 0 | 0 | 0 |
| Conjunctival deposit | 1 (0.15) | 0 | 0 | 0 |
| Dry eye | 1 (0.15) | 0 | 0 | 0 |
| Keratitis | 1 (0.15) | 0 | 0 | 0 |
| Ocular hypertension | 1 (0.15) | 0 | 0 | 0 |
| Ulcerative keratitis | 1 (0.15) | 0 | 0 | 0 |
| **Investigations** | 4 (0.62) | 1 (0.15) | 1 (0.15) | 0 |
| Intraocular pressure increased | 4 (0.62) | 1 (0.15) | 1 (0.15) | 0 |
| **Injury, poisoning, and procedural complications** | 1 (0.15) | 1 (0.15) | 1 (0.15) | 1 (0.15) |
| Lenticular injury | 1 (0.15) | 1 (0.15) | 1 (0.15) | 1 (0.15) |
| **Infections and infestations** | 1 (0.15) | 1 (0.15) | 0 | 0 |
| Dacryocystitis | 1 (0.15) | 1 (0.15) | 0 | 0 |
| **Nervous system disorders** | 1 (0.15) | 0 | 0 | 0 |
| Facial paralysis | 1 (0.15) | 0 | 0 | 0 |
| **Non-ocular events, total** | 18 (2.79)[a] | 15 (2.32)[a] | 4 (0.62) | 3 (0.46) |
| **Nervous system disorders** | 6 (0.93) | 4 (0.62) | 3 (0.46) | 2 (0.31) |
| Cerebral infarction | 4 (0.62) | 4 (0.62) | 2 (0.31) | 2 (0.31) |
| Facial paralysis | 1 (0.15) | 0 | 1 (0.15) | 0 |
| Migraine | 1 (0.15) | 0 | 0 | 0 |
| **Infections and infestations** | 4 (0.62) | 4 (0.62) | 0 | 0 |
| Gangrene | 2 (0.31) | 2 (0.31) | 0 | 0 |
| Peritonitis | 1 (0.15) | 1 (0.15) | 0 | 0 |
| Sepsis | 1 (0.15) | 1 (0.15) | 0 | 0 |
| **Renal and urinary disorders** | 3 (0.46) | 3 (0.46) | 0 | 0 |
| Chronic kidney disease | 3 (0.46) | 3 (0.46) | 0 | 0 |
| **Cardiac disorders** | 2 (0.31) | 2 (0.31) | 1 (0.15) | 1 (0.15) |
| Myocardial infarction | 1 (0.15) | 1 (0.15) | 1 (0.15) | 1 (0.15) |
| Cardiac failure | 1 (0.15) | 1 (0.15) | 0 | 0 |
| **Injury, poisoning, and procedural complications** | 2 (0.31) | 2 (0.31) | 0 | 0 |
| Injury | 1 (0.15) | 1 (0.15) | 0 | 0 |
| Road traffic accident | 1 (0.15) | 1 (0.15) | 0 | 0 |
| **Investigations** | 2 (0.31) | 0 | 0 | 0 |
| Glycosylated hemoglobin increased | 2 (0.31) | 0 | 0 | 0 |
| **Neoplasms benign, malignant, and unspecified (including cysts and polyps)** | 1 (0.15) | 1 (0.15) | 0 | 0 |
| Colon cancer | 1 (0.15) | 1 (0.15) | 0 | 0 |
| **Blood and lymphatic system disorders** | 1 (0.15) | 0 | 0 | 0 |
| Anemia | 1 (0.15) | 0 | 0 | 0 |

[a]Including patients with multiple events

*AE* adverse event; *SAE* serious adverse event; *ADR* adverse drug reaction; *SADR* serious adverse drug reaction

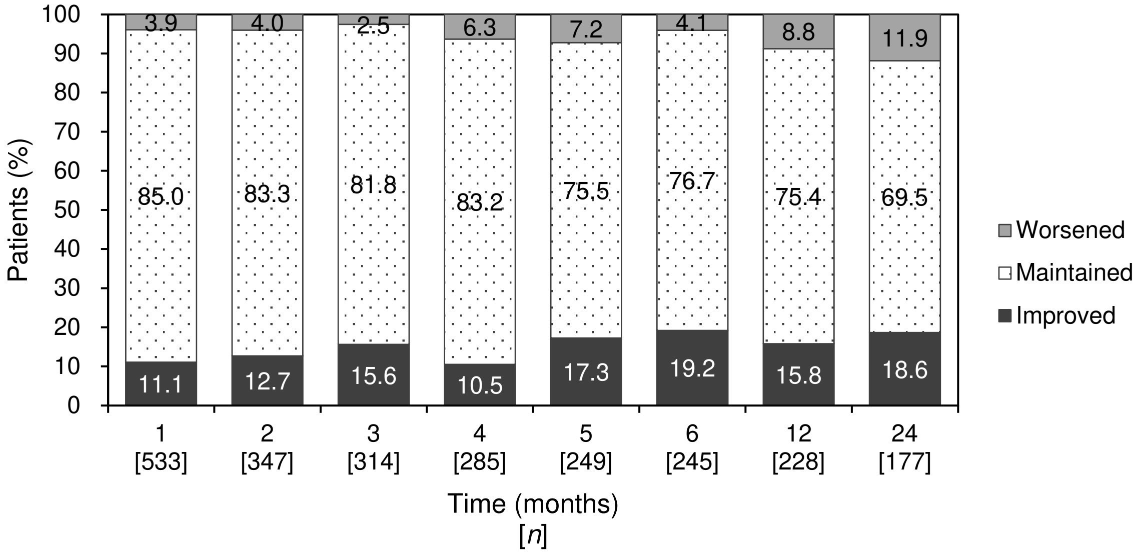


**Supplementary Information 7** Proportions of patients with improved, maintained, or worse BCVA. The logMAR BCVA change from baseline was classified as follows: “improved”, logMAR BCVA change ≤ −0.3; “maintained”, logMAR BCVA change between −0.3 and 0.3; and “worse”, logMAR BCVA change ≥ 0.3.

*BCVA* best-corrected visual acuity; *logMAR* logarithm of the minimum angle of resolution

**a**

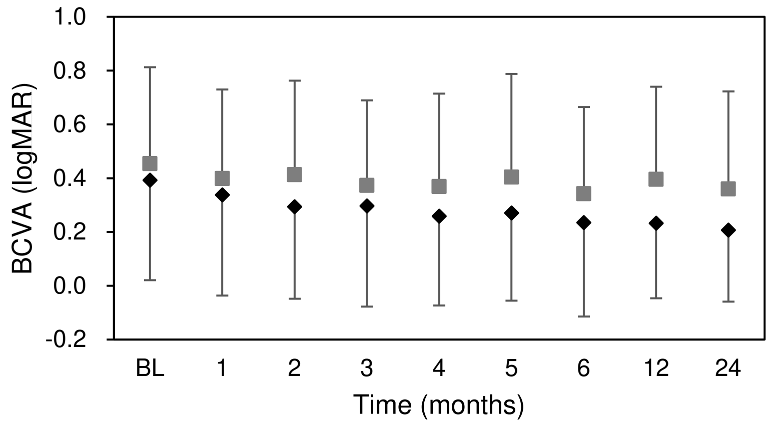


| | | BL | 1 | 2 | 3 | 4 | 5 | 6 | 12 | 24 |
|---|---|---|---|---|---|---|---|---|---|---|
| ■ With previous treatment | BCVA (logMAR) | 0.454 | | | | | | 0.342 | 0.396 | 0.360 |
| | [n] | [459] | [395] | [252] | [232] | [211] | [187] | [182] | [167] | [132] |
| ◆ Without Previous treatment | BCVA (logMAR) | 0.393 | | | | | | 0.235 | 0.233 | 0.207 |
| | [n] | [155] | [133] | [91] | [80] | [71] | [59] | [60] | [59] | [42] |

**b**

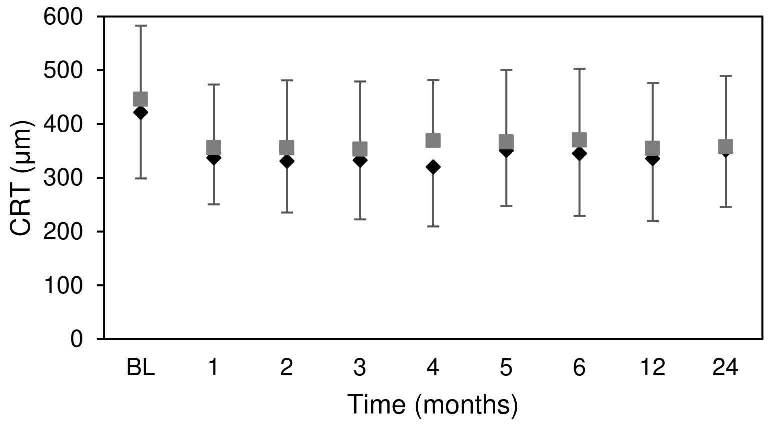


| | | BL | 1 | 2 | 3 | 4 | 5 | 6 | 12 | 24 |
|---|---|---|---|---|---|---|---|---|---|---|
| ■ With previous treatment | CRT (μm) | 446.2 | | | | | | 370.4 | 354.9 | 357.8 |
| | [n] | [343] | [301] | [207] | [193] | [161] | [139] | [146] | [134] | [109] |
| ◆ Without previous treatment | CRT (μm) | 421.7 | | | | | | 345.4 | 335.9 | 351.7 |
| | [n] | [98] | [85] | [66] | [58] | [43] | [36] | [39] | [43] | [30] |

**Supplementary Information 8** Subgroup analysis based on the presence/absence of previous treatment. (a) LogMAR BCVAs and numbers of patients during the 24-month study period. (b) CRTs (μm) and numbers of patients during the 24-month study period. The mean and standard deviation are indicated with markers and whiskers, respectively.

*BCVA* best-corrected visual acuity; *BL* baseline; *CRT* central retinal thickness; *logMAR* logarithm of the minimum angle of resolution

**a**

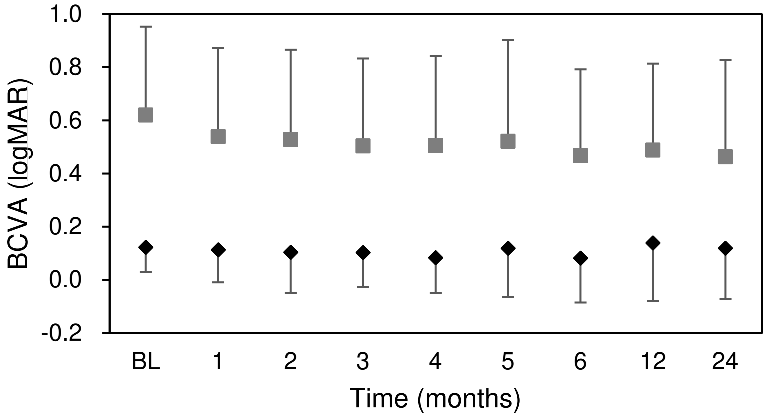


| | | BL | 1 | 2 | 3 | 4 | 5 | 6 | 12 | 24 |
|---|---|---|---|---|---|---|---|---|---|---|
| ■ BL decimal | BCVA (logMAR) | 0.620 | | | | | | 0.467 | 0.488 | 0.463 |
| BCVA ≤ 0.5 | [n] | [393] | [337] | [225] | [194] | [173] | [154] | [147] | [139] | [104] |
| ◆ BL decimal | BCVA (logMAR) | 0.123 | | | | | | 0.082 | 0.139 | 0.119 |
| BCVA > 0.5 | [n] | [229] | [196] | [122] | [120] | [112] | [95] | [98] | [89] | [73] |

**b**

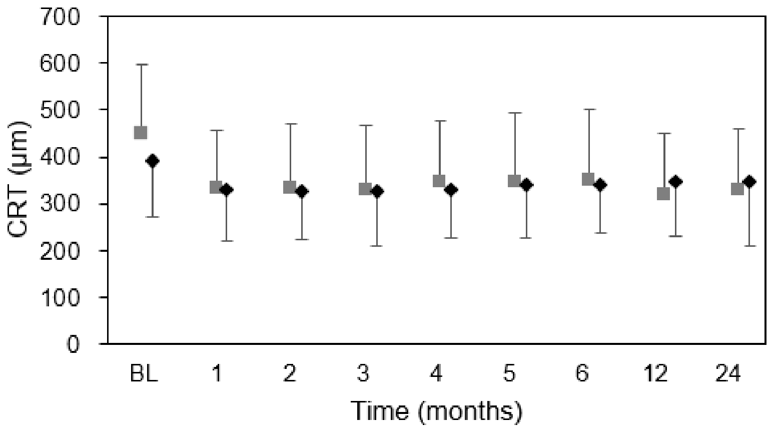


| | | BL | 1 | 2 | 3 | 4 | 5 | 6 | 12 | 24 |
|---|---|---|---|---|---|---|---|---|---|---|
| ■ BL decimal | CRT (μm) | 464.0 | | | | | | 369.1 | 341.3 | 349.8 |
| BCVA ≤ 0.5 | [n] | [268] | [234] | [170] | [146] | [117] | [108] | [111] | [107] | [81] |
| ◆ BL decimal | CRT (μm) | 405.4 | | | | | | 357.0 | 361.9 | 363.4 |
| BCVA > 0.5 | [n] | [176] | [155] | [106] | [107] | [87] | [69] | [76] | [71] | [59] |

**Supplementary Information 9** Subgroup analysis based on the baseline decimal BCVA. (a) LogMAR BCVAs and numbers of patients during the 24-month study period. (b) CRTs (μm) and numbers of patients during the 24-month study period. The mean and standard deviation are indicated with markers and whiskers, respectively.

*BCVA* best-corrected visual acuity; *BL* baseline; *CRT* central retinal thickness; *logMAR* logarithm of the minimum angle of resolution
